# Analysis of Motor Function in the Tg4-42 Mouse Model of Alzheimer’s Disease

**DOI:** 10.3389/fnbeh.2019.00107

**Published:** 2019-05-17

**Authors:** Jannek M. Wagner, Marius E. Sichler, Eva M. Schleicher, Timon N. Franke, Caroline Irwin, Maximilian Johannes Löw, Nicola Beindorff, Caroline Bouter, Thomas A. Bayer, Yvonne Bouter

**Affiliations:** ^1^Division of Molecular Psychiatry, Department of Psychiatry and Psychotherapy, University Medical Center Göttingen, Georg-August-University, Göttingen, Germany; ^2^Berlin Experimental Radionuclide Imaging Center, Charité – University Medicine Berlin, Berlin, Germany; ^3^Department of Nuclear Medicine, University Medical Center Göttingen, Georg-August-University, Göttingen, Germany

**Keywords:** motor function, cerebellum, Alzheimer, transgenic mice, behavior, FDG-PET

## Abstract

Alzheimer’s disease (AD) is a neurodegenerative disorder and the most common form of dementia. Hallmarks of AD are memory impairments and cognitive deficits, but non-cognitive impairments, especially motor dysfunctions are also associated with the disease and may even precede classic clinical symptoms. With an aging society and increasing hospitalization of the elderly, motor deficits are of major interest to improve independent activities in daily living. Consistent with clinical findings, a variety of AD mouse models develop motor deficits as well. We investigated the motor function of 3- and 7-month-old Tg4-42 mice in comparison to wild-type controls and 5XFAD mice and discuss the results in context with several other AD mouse model. Our study shows impaired balance and motor coordination in aged Tg4-42 mice in the balance beam and rotarod test, while general locomotor activity and muscle strength is not impaired at 7 months. The cerebellum is a major player in the regulation and coordination of balance and locomotion through practice. Particularly, the rotarod test is able to detect cerebellar deficits. Furthermore, supposed cerebellar impairment was verified by ^18^F-FDG PET/MRI. Aged Tg4-42 mice showed reduced cerebellar glucose metabolism in the ^18^F-FDG PET. Suggesting that, deficits in coordination and balance are most likely due to cerebellar impairment. In conclusion, Tg4-42 mice develop motor deficits before memory deficits, without confounding memory test. Thus, making the Tg4-42 mouse model a good model to study the effects on cognitive decline of therapies targeting motor impairments.

## Introduction

Alzheimer’s disease (AD) is a neurodegenerative disorder and the most common form of dementia. The main clinical symptoms of AD are memory impairments and cognitive deficits. However, non-cognitive impairments especially motor dysfunctions are also associated with the disease. The observed motor deficits range from impaired balance and gait changes to disturbed activity levels (Larson et al., 1992; O’Keeffe et al., 1996; Pettersson et al., 2002; Camicioli et al., 2006).

In AD patients, motor impairments may even precede the clinical symptoms, providing a link between motor function and the development of AD (Buchman and Bennett, 2011).

Motor signs (MOSIs) decline with age and severity of the disease. The most affected MOSIs are speech, facial expression, rigidity, posture, gait, bradykinesia, and less frequently tremor (Scarmeas et al., 2004).

From mild to moderate AD the decline in motor abilities is stronger than from moderate to severe AD and associated with an increased risk of falls (Zidan et al., 2012). Slowing in fine motor dexterity seems to depend on the severity of the disease and is associated with functional decline in the active daily living (de Paula et al., 2016). In the ‘Sydney Older Persons Study’ a combination of cognitive decline and motor deficits was investigated over a 6-year period. Motor slowing and gait abnormalities resulted in poorer outcome of patients with dementia. Patients with decreased motor abilities were more likely to have severe cognitive impairments and even died earlier (Waite et al., 2005). Another study showed that gait abnormalities in aged patients without the diagnosis of dementia at baseline were related to lower cognitive performance and an increased incidence of mild cognitive impairment (MCI) or dementia (Beauchet et al., 2014). Wilson et al. (2000) showed a link between progression in parkinsonism (bradykinesia, gait disorder/postural reflex impairment, rigidity, tremor) and the progression of cognitive decline in AD patients. Furthermore, rigidity has been shown to be linked to death as well and to increased hospitalization in AD patients (Lopez et al., 1997).

Consistent with clinical findings, a variety of AD mouse models develop motor deficits (Kobayashi and Chen, 2005; Wirths and Bayer, 2008) (Table 1). There are several well established behavioral tests to assess motor function, coordination and balance in rodents such as the rotarod test, balance beam task, string suspension task, inverted grip strength task and the Open Field task for locomotor activity (Brooks and Dunnett, 2009; Deacon, 2013).

**Table 1:** Motor function of different AD mouse models.

Mouse model	References	Test age	Sex	Open Field	Rotarod	Balance beam walk	String susp. task	Grip strength tasks
**J20** APP_SweK670N/M671L__+__IndV 717F_ (Mucke et al., 2000)	Harris et al., 2010	1) 2–3 m	♂	↑	–	–	–	–
		2) 5–7 m	♂	↑	–	–	–	–
	Chang et al., 2015	1) 5 m	–	ns	ns	–	–	–

**Tg2576**APP695_SweK670N/M671L_ (Hsiao et al., 1996)	King and Arendash, 2002	1) 3 m	♀♂	↑	–	↓	ns	–
		2) 9 m	♀♂	ns	–	ns	ns	–
		3) 14 m	♀♂	ns	–	↓	↓	–
		4) 19 m	♀♂	ns	–	↓	↓	–
	Dineley et al., 2002	1) 5 m	♀♂	ns	ns	–	–	–
		2) 9 m	♀♂	ns	ns	–	–	–
	Perucho et al., 2010	1) 12 m	♂	ns	ns	–	–	–

**APP23**APP751_SweK670N/M671L_ (Sturchler-Pierrat et al., 1997)	van Dam et al., 2003	1) 6–8w	♂	ns	ns	–	–	–


		2) 3 m	♂	ns	↓	–	–	–
		3) 6 m	♂	↓	↓	–	–	–
	Lalonde et al., 2005a	1) 24 m	♀	ns	↑	ns	↑	–

**APP/PS1**APP_SweK670N/M671L_/PS1_ΔE9_ (Jankowsky et al., 2001)	Kuwabara et al., 2014	1) 3 m	♀♂	–	↓	ns	–	–
		2) 5–6 m	♂	–	↓	ns	–	–
	Singh et al., 2017	1) 4 m	♀♂	–	ns	–	–	–
		2) 8 m	♀♂	–	ns	–	–	–
		3) 12 m	♀♂	–	ns	–	–	–
	Lalonde et al., 2004	1) 7 m	♀♂	ns	ns	ns	ns	ns

**APP+PS1**APP_SweK670N/M671L_ + PS1_M146L_ (Holcomb et al., 1999)	Holcomb et al., 1999	1) 3 m	♀♂	–	–	–	ns	–
		2) 6 m	♀♂	–	–	–	ns	–
		3) 9 m	♀♂	–	–	–	ns	–
	Arendash et al., 2001	1) 5–7 m	♀♂	ns	–	↓	ns	–
		2) 15–17 m	♀♂	↑	–	↓	↓	–
	Sadowski et al., 2004	1) 8 m	–	ns	–	ns	–	–
		2) 22 m	–	ns	–	ns	–	–
	Ewers et al., 2006	1) 12 m	♀♂	–	↓	–	–	–

**APP/PS1KI**APP_NLh/NLh_ × PS1_P264L/P264L_ (Flood et al., 2002)	Webster et al., 2013	1) 7 m	♀♂	ns	ns	ns	–	ns
		2) 11 m	♀♂	ns	ns	ns	–	ns
		3) 15 m	♀♂	ns	ns	ns	–	ns
		4) 24 m	♀♂	ns	ns	ns	–	ns

**5XFAD**APP_sweK670N/M671, FloI716V,__LonV 717I_ + PS1_M146/L286V_ (Oakley et al., 2006)	Jawhar et al., 2012	1) 3 m	♀	–	–	ns	ns	–
		2) 6 m	♀	–	–	ns	ns	–
		3) 9 m	♀	ns	–	↓	↓	–
		4) 12 m	♀	ns	–	↓	↓	–
	O’Leary et al., 2018a	1) 3–4 m	♀♂	ns	ns	ns	ns	ns
		2) 6–7 m	♀♂	♀♂	ns	ns	♂↓	↓
		3) 9–10 m	♀♂	ns	↓	↓	ns	ns
		4) 12–13 m	♀♂	↓	↓	↓	♂↓	↓
		5) 15–16 m	♀♂	↓	↓	↓	↓	↓
	Shukla et al., 2013	1) 6 m	♀♂	ns	ns	–	–	–
		2) 9 m	♀♂	–	ns	–	–	–
		3) 12 m	♀♂	–	↓	–	–	–
	current study	1) 3 m	♀	ns	ns	ns	ns	ns
		2) 7 m	♀	↓	ns	ns	ns	ns

**3xTg** APP_SweK670M/N671L_, PS1_M136V_, MAPT_P301L_ (Oddo et al., 2003)	Oore et al., 2013	1) 2 m	♀♂	–	↑	–	–	–
		2) 6 m	♀♂	–	↑	–	–	–
		3) 9 m	♀♂	–	↑	–	–	–
		4) 12 m	♀♂	–	↑	–	–	–
		5) 15 m	♀♂	–	↑	–	–	–
	Stover et al., 2015	1) 6 m	♀♂	–	↑	ns	ns	↓
	Garvock-de Montbrun et al., 2019	1) 16 m	♀♂	–	↑	ns	ns	ns
	Filali et al., 2012	1) 12–14 m	♀	↓	↓	–	–	–
	Gulinello et al., 2009	1) 15–18 m	♀♂	↓	–	ns	–	–

**TBA 42** Aβ_pE3-42_ (Wittnam et al., 2012)	Meissner et al., 2014	1) 3 m	♀♂	–	–	ns	↓	ns
		2) 6 m	♀♂	–	–	↓	↓	↓
		3) 12 m	♀♂	–	–	↓	↓	↓
	Wittnam et al., 2012	1) 3 m	♀	–	–	ns	–	–
		2) 6 m	♀	–	–	ns	–	–
		3) 12 m	♀	–	–	↓	–	–
	Lopez-Noguerola et al., 2018	1) 3–4 m	♀♂	–	–	ns	ns	ns
		2) 5–6 m	♀♂	–	–	↓	↓	↓

**Tg4-42** Aβ_4-42_ (Bouter et al., 2013)	Lopez-Noguerola et al., 2018	1) 3–4 m	♀♂	–	–	ns	ns	ns
		2) 5–6 m	♀♂	–	–	ns	ns	ns
	Current study	1) 3 m	♀	ns	↓	ns	ns	ns
		2) 7 m	♀	ns	↓	↓	ns	ns

*w, weeks; m, months; ns, not significant.*

While the exact etiology of AD is still not fully understood, considerable evidence points to amyloid-beta peptides (Aβ) as a key player in the pathogenesis of AD. According to the amyloid cascade hypothesis, Aβ-plaques seem to play a causative role in the pathogenesis of AD (Hardy and Higgins, 1992). More recent data claim that, truncated and modified Aβ variants play an important role next to full-length Aβ. Especially N-truncated forms of Aβ enhance aggregation and neurotoxicity (Pike et al., 1995; Bayer and Wirths, 2014). Among the different species, Aβ beginning with phenylalanine at position 4 is particularly abundant in the brain of AD patients (Masters et al., 1985; Portelius et al., 2010). The transgenic mouse model Tg4-42 expresses exclusively intraneuronal Aβ4-42 without human amyloid precursor protein (APP) overexpression (Bouter et al., 2013). Intracellular Aβ accumulation is accompanied by micro- and astrogliosis that is most abundant in the hippocampus of these mice. Tg4-42 mice develop severe synaptic impairments and neuron loss especially in the CA1 region of the hippocampus. Furthermore, Tg4-42 mice develop age-dependent behavior and memory deficits albeit without plaque formation (Bouter et al., 2013, 2019; Dietrich et al., 2013).

The aim of the current study was to extend previous findings on the Tg4-42 model by examining motoric abilities and compare these results with the widely used 5XFAD mouse model. We investigated mice at 3 months, with no known memory deficits in comparison with 7-month-old mice, which already present strong memory deficits. Furthermore, we discuss the results in the context of other, well-studied AD models, to facilitate model selection for further research on the causes of motor impairments related to AD and the development of possible therapies.

## Materials and Methods

### Transgenic Mice

The generation of Tg4-42 mice has been described previously (Bouter et al., 2013). Briefly, Tg4-42 mice express human Aβ4-42 fused to the murine thyrotropin releasing hormone signal peptide under the control of the neuronal Thy-1 promoter. Tg4-42 mice were generated and maintained on a C57Bl/6J genetic background. Only homozygous Tg4-42 mice were used in this study.

The double transgenic 5XFAD model (Jackson Laboratories, Bar Harbor, ME, United States) over-expresses the 695 amino acids isoform of the human amyloid precursor protein (APP695) carrying the Swedish, London, and Florida mutations under the control of the murine Thy-1 promoter. In addition, human presenilin-1 (PSEN-1) carrying the M146L/L286V mutations is also expressed under the control of the murine Thy-1 promoter in 5XFAD mice (Oakley et al., 2006). 5XFAD mice used in the current study were kept on a C57Bl/6J genetic background (Jawhar et al., 2012). Wild-type littermates served as age-matched control animals. In the current study, only female mice were used.

All animals were handled according to the guidelines of the ‘Society for Laboratory Animals Science’ (GV-SOLAS) and the guidelines of the ‘Federation of European Laboratory Animal Science Association’ (FELASA). All experiments were approved by the ‘Lower Saxony State Office for Consumer Protection and Food Safety’ (LAVES).

Mice were kept on a 12 h/12 h inverted light cycle and behavior experiments were performed during the dark phase. Mice were subjected to a battery of behavior tests at 3 and 7 months of age to assess possible motor deficits. Weight was monitored as part of a general physical assessment.

### Paw-Clasping Test

The clasping test was used to test for functional impairments (Jawhar et al., 2012). Each mouse was suspended by their tail for 30 s to provoke a clasping phenotype. Healthy mice try to escape the grip by twisting their body and kicking their paws and therefore do not show any clasping phenotype. Clasping behavior was scored on a scale from zero to three: 0 = no clasping behavior, 1 = fore paws clasping, 2 = one hind paw and fore paws clasping, 3 = clasping of all paws (Miller et al., 2008).

### String Suspension

Grip strength and general motor coordination were analyzed using the string suspension task as described previously (Jawhar et al., 2012). In brief, mice were permitted to grasp a cotton string with their fore paws and were then released. Their ability to climb across the string was assessed using a 0 to 5 rating score: 0 = falls of string; 1 = hangs onto string by fore- or hind paws; 2 = hangs onto string by fore- or hind paws and attempts to climb onto string; 3 = hangs onto string by all four paws but no lateral movement; 4 = hangs onto string using all four paws and tail and moves laterally; 5 = escapes to the edge of string and touches wooden support beam (Moran et al., 1995). Each animal performed three 60-s trials throughout 1 day with a minimum of 30 min between the trials. The average score of all three trials was taken for each mouse.

### Balance Beam

Fine motor coordination and balance of mice were assessed using the balance beam test as previously described (Jawhar et al., 2012). The test essentially examines the ability of a mouse to remain upright and to walk on the relatively narrow and elevated beam to one of the platforms. During a single day of testing each mouse was given three 60-s trials with a minimum of 10 min between the trials. The average time of all three trials was taken as the score for each mouse. A test trial was given to familiarize the mouse with the beam. For each trial, the mouse was placed in the center of the beam facing one of the platforms and then released. The latency to fall from the beam was recorded. If a mouse escaped to one of the platforms or remained on the beam for the entire trial, the maximum time of 60 s was given.

### Inverted Grip Task

Neuromuscular abilities and muscle strength were tested with the inverted grip test as previously described (Wirths et al., 2008). Mice were placed on a metal grid and turned upside down 30 cm elevated above a padded surface. Latency to fall within 60 s was measured in one single trial.

### Rotarod

Motor performance and motor learning were tested using the rotarod (TSE Systems, Germany). Testing consists of four trials per day for two consecutive days with intertrial intervals of 10–15 min. Each mouse was placed on the rod, which accelerated from 4 to 40 rpm. over the trial time of 300 s. Trials were terminated when animals fell off or the maximum time was reached. Up to five mice were tested simultaneously, separated by black plastic walls. Mice were taken out of the apparatus when the last mouse fell. Latency to fall served as an indicator of motor coordination.

### ^18^F-FDG PET/MRI

^18^F-fluoro-deoxy-glucose positron emission tomography/magnetic resonance image (^18^F-FDG-PET/MRI) acquisition and analysis were used to evaluate brain glucose metabolism in the cerebellum of 7-month-old Tg4-42 mice and 5XFAD mice. Female Tg4-42 (*n* = 5), 5XFAD (*n* = 3) and wild-type C57Bl/6J (*n* = 5) control mice were fasted overnight and blood glucose levels were measured. As previously described 9–21 MBq (mean 15.76 MBq) of ^18^F-FDG were administered intravenously into a tail vein with a maximum volume of 200 μl (Bouter et al., 2019). Mice were anesthetized with isoflurane supplemented with oxygen during the scans and were awake during the uptake period. After an uptake period of 45 min, PET imaging was performed on a small animal 1 Tesla nanoScan PET/MRI (Mediso, Hungary). During the scan mice were placed on a 37°C heated bed. Respiration rate was monitored constantly during the scans. PET scans were performed for 20 min. MRI-based attenuation correction was conducted (matrix 144 × 144 × 163 with a voxel size of 0.5 × 0.5 × 0.6 mm^3^, TR: 15 ms, TE 2.032 ms and a flip angle of 25°) and the PET images were reconstructed with the following parameters: matrix 136 × 131 × 315 with a voxel size of 0.23 × 0.3 × 0.3 mm^3^. Image analysis was performed using PMOD v3.9 (PMOD Technologies, Switzerland). A predefined mouse brain atlas template was used to analyze different brain areas including the cerebellum (Cb). Corresponding PET images were matched to the MRI and statistics within the cerebellum volume of interest (VOI) in kBq/cc were generated. Standardized uptake value (SUV) was calculated [SUV = tissue activity concentration average (KBq/cc) × body/weight (g)/injected dose (kBq)] for semi-quantitative analysis and SUV values were corrected for blood glucose levels [SUVGlc = SUV × blood glucose level (mg/dl)].

### Statistical Analysis

Differences between groups were tested with one-way analysis of variance (ANOVA) or two-way ANOVA followed by analysis of Bonferroni multiple comparison indicated. All data are given as mean ± standard error of the mean (SEM). Significance levels are given as follows: ^∗∗∗^*p* < 0.001; ^∗∗^*p* < 0.01; ^∗^*p* < 0.05. GraphPad Prism version 6.07 for Windows (GraphPad Software, San Diego, CA, United States) was used for all calculations.

## Results

### Normal Body Weight of Tg4-42 and 5XFAD Mice

**FIGURE 1:**
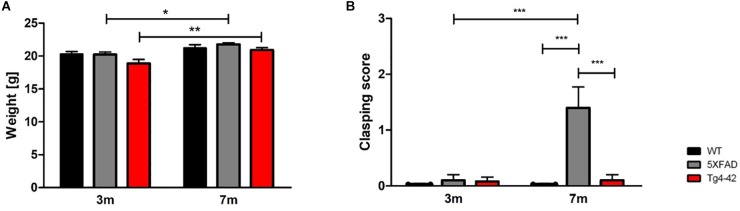
General physical assessment of Tg4-42 and 5XFAD mice. **(A)** 3- and 7-month-old Tg4-42 and 5XFAD mice displayed normal body weight compared to aged-matched wild-type mice. **(B)** Wild-type and Tg4-42 mice showed no clasping phenotype during the tail suspension task regardless of age. 5XFAD mice showed a clasping phenotype at 7 months of age. Two-way repeated measures ANOVA, ^∗^*p* < 0.05, ^∗∗^*p* < 0.01, ^∗∗∗^*p* < 0.001; *n* = 12 per group; data presented as mean ± SEM; WT, wild-type; m, months.

The body weight of all mice was taken at 3 and 7 months of age. Tg4-42 and 5XFAD mice demonstrated no altered body weight compared to wild-type mice at the tested time points [Figure 1A, two-way ANOVA, *genotype: F*(2,66) = 3.744, *p* = 0.0688]. Statistically significant was the gain of weight of Tg4-42 and 5XFAD mice, but although wild-type mice gained some weight as well, this effect was not statistically significant [Figure 1A, two-way ANOVA, *age: F*(1,66) = 18.55, *p* < 0.0001, *post hoc* analysis Tg4-42: *p* < 0.01, 5XFAD: *p* < 0.05].

### Clasping Phenotype in 7-Month-Old 5XFAD Mice

At 3 months none of the tested animals showed any clasping phenotype. However, 5XFAD mice develop an age-dependent clasping phenotype [Figure 1B, two-way ANOVA, *age: F*(1,66) = 10.61, *p* = 0.0019; *genotype: F*(2,66) = 12.12, *p* < 0.001]. Tg4-42 mice did not show a clasping behavior at 7 months and they tried to escape the grip by twisting their body and kicking their paws similar to wild-type animals.

### Impaired Sensorimotor Function in Tg4-42 Mice

**FIGURE 2:**
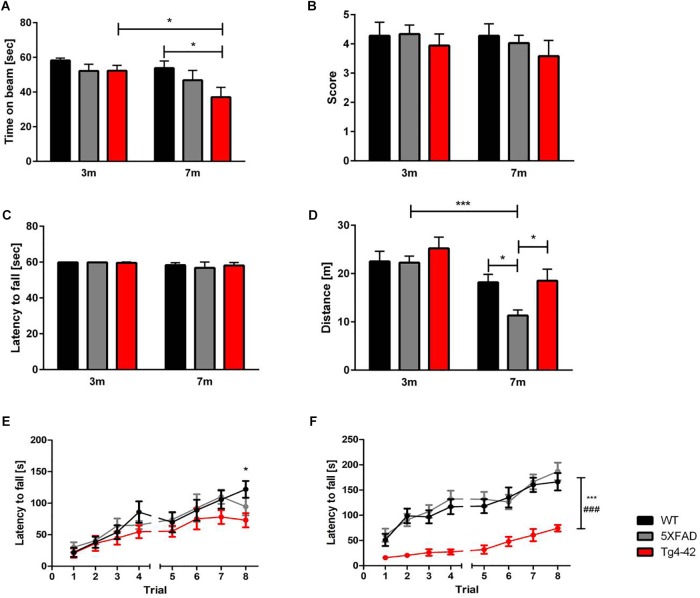
Motor deficits in aged Tg4-42 mice. **(A)** Tg4-42 mice showed age-dependent motor deficits in the balance beam. 5XFAD mice displayed no deficits in the balance beam. Tg4-42 and 5XFAD mice performed similar to same-aged wild-type control mice in the **(B)** string suspension task and **(C)** inverted grip task. **(D)** 5XFAD mice displayed an age-dependent decreased distance traveled in the Open Field task. Seven-month-old 5XFAD mice traveled significantly less than same-aged Tg4-42 and wild-type mice. **(E)** Young Tg4-42 and 5XFAD mice showed a similar latency to fall on the accelerating rotarod on trials 1–7. On the last trial of the test Tg4-42 mice performed worse than same-aged wild-type mice. **(F)** Aged Tg4-42 mice showed a decreased performance on the accelerating rotarod compared to same-aged wild-type and 5XFAD mice. Two-way repeated measures ANOVA, vs. 5XFAD ^###^*p* < 0.001; vs. WT ^∗∗∗^*p* < 0.001; ^∗^*p* < 0.05; *n* = 9–15 per group, data presented as mean ± SEM; WT, wild-type; m, month.

Fine motor coordination and balance of mice can be assessed by the balance beam task (Hau and Schapiro, 2002; Luong et al., 2011). At the age of 3 months none of the tested animals showed impairments in the balance beam task. However, 7-month-old Tg4-42 mice spent significantly less time on the beam compared to same-aged wild-type animals [Figure 2A, two-way ANOVA, *genotype: F*(2,66) = 3.702; *p* < 0.05]. Tg4-42 mice showed an age-dependent decline in balance and motor coordination [Figure 2A, two-way ANOVA, Tg4-42 *age: F*(1,66) = 5.904, *p* = 0.0178; *post hoc* analysis Tg4-42: *p* < 0.05]. In contrast, aged 5XFAD mice performed similar to wild-type controls.

### Unaltered Grip Strength in 5XFAD and Tg4-42 Mice

The string suspension task evaluates motor coordination, grip and muscle strength of mice (Hullmann et al., 2017), whereas the inverted grip task assesses muscle strength (Deacon, 2013). Neither 5XFAD, nor Tg4-42 mice showed a lower grip strength in any of these tests compared to wild-type control mice [Figure 2B,C, two-way ANOVA, string suspension *genotype: F*(2,66) = 0.9058, *p* = 0.4092, *age: F*(1,66) = 0.450, *p* = 0.5047; inverted grip *genotype: F*(1,66) = 2.435, *p* = 0.1234, *age*: *F*(1,66) = 2.435, *p* = 0.1234].

### Decreased Locomotor Activity of 5XFAD, but Not Tg4-42 Mice in the Open Field Task

To measure general locomotor activity, mice were tested in an Open Field task (Seibenhener and Wooten, 2015). Tg4-42 mice traveled the same distance as wild-type controls, showing no impaired locomotor activity at any tested age. On the other hand, 5XFAD mice traveled less, showing an age-dependent decreased in locomotor activity [Figure 2D, two-way ANOVA, *age*: *F*(1,71) = 22.33, *p* < 0.001; *genotype*: *F*(2,71) = 3.706, *p* = 0.0294].

### Impaired Motor Learning Skills in Tg4-42 Mice

The rotarod task assesses motor skill learning abilities and coordination (Buitrago et al., 2004; Deacon, 2013). Performance of 5XFAD mice did not differ from wild-type controls at 3 or 7 months of age. While young Tg4-42, 5XFAD and WT mice showed an overall improved motor learning over the training trials [Figure 2E, two-way ANOVA, *trials*: *F*(7,210) = 20.18, *p* < 0.0001], Tg4-42 displayed a shorter latency to fall on the last trial compared to same-aged wild-type animals [Figure 2E, two-way ANOVA, *genotype*: *F*(2,30) = 1.114, *p* = 0.3413; *post hoc* analysis Tg4-42 *trial 8*: *p* < 0.05]. At 7 months of age Tg4-42 mice showed decreased motor learning and performed significantly worse than wild-type and 5XFAD animals [Figure 2F, two-way ANOVA, *genotype: F*(2,41) = 33.00, *p* < 0.0001].

### Decreased Metabolic Activity in the Cerebellum of Aged Tg4-42 Mice

**FIGURE 3:**
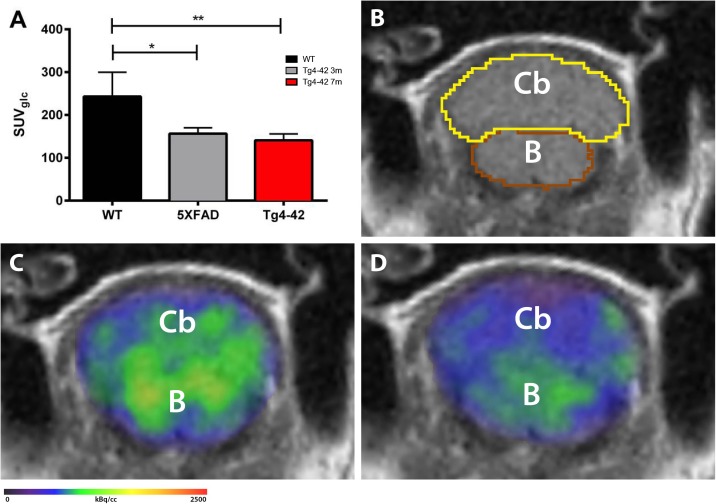
^18^F-FDG-PET shows decreased metabolic activity in the cerebellum of 7-month-old Tg4-42 and 5XFAD mice. **(A)** Quantification of ^18^F-FDG uptake in the cerebellum. ^18^F-FDG-uptake in the cerebellum was significantly reduced in aged Tg4-42 and 5XFAD mice compared to wild-type. **(B)** Magnetic resonance image (MRI; coronal view) with volumes of interest (VOIs) of the mouse brain atlas MRIs of each mouse. **(C)** Fused ^18^F-FDG-PET/MRI of a wild-type mouse in coronal view. **(D)** Fused ^18^F-FDG-PET/MRI of a 7-month-old Tg4-42 mouse in coronal view with distinctly lower FDG uptake compared to the wild-type mouse. One-way-ANOVA; ^∗∗^*p* < 0.01; ^∗^*p* < 0.05; WT, wild-type; m, months; Cb, cerebellum; B, brainstem.

^18^F-FDG-PET/MRI was used to determine cerebellar glucose metabolism. Quantitative analysis of FDG-uptake was performed using a mouse brain atlas and blood glucose corrected SUV values (SUVglc) were measured within a predefined cerebellum VOI (Figure 3B). Seven-month-old Tg4-42 and 5XFAD mice showed significantly decreased ^18^F-FDG uptake in the cerebellum compared to wild-type mice [Figure 3A,C,D, one-way-ANOVA, *F*(2,11) = 2.007, *p* = 0.0018; *post hoc* analysis WT vs. Tg4-42: *p* < 0.01; WT vs. 5XFAD: *p* < 0.05].

## Discussion

Impairments of motor abilities are an important phenotype in the progression of AD. Several studies show a decline in motor function throughout the progression of the disease in patients (Zidan et al., 2012; Beauchet et al., 2014; de Paula et al., 2016). Furthermore, motor impairments can be useful in the prediction of the onset as well as the outcome of AD (Waite et al., 2005). In an aging society, maintaining motor performance in AD patients should be of major interest to help to facilitate independence in daily living and activity (Yan and Zhou, 2009). Assessing motor deficits through physical exercise in preclinical stages of AD could delay or decline the development of AD as shown previously (Larson et al., 2006). Thus, motor deficits are an important symptom to study in AD.

Similar to AD patients, most mouse models mimicking AD show motor impairments as the disease progresses. This study compared motor behavior of young and aged Tg4-42 mice with their age-matched wild-type controls and compared the results with other AD mouse models. Motoric performance of AD mice ranges from unaltered and impaired to improved motor performance. There are several tests to assess motor abilities in mice, but protocols and experimental apparatus differ highly between laboratories, complicating the comparison of results. These differences in behavior tests should always be taken into consideration (Table 1).

Weight can severely influence the outcome of behavior studies. Therefore, the animal’s weight should always be taken into consideration especially when analyzing motor performance in mice. Previous studies showed that body weight significantly correlates with the performance of mice in the rotarod task, a test widely used to assess grip strength, motor coordination and balance (Brown and Wong, 2007; Shiotsuki et al., 2010). The weight of young and aged Tg4-42 mice was comparable to same-aged wild-type and 5XFAD mice in this study. In contrast, weight loss has previously been observed in several other AD mouse models. A reduction in the gain of body weight has been described especially in mouse models overexpressing APP (Lalonde et al., 2005a; Pugh et al., 2007; Alexandru et al., 2011). For example, 9-month-old 5XFAD mice showed a significantly reduced body weight in comparison to their wild-type littermates (Jawhar et al., 2012). Interestingly, epidemiologic studies showed that weight loss is often associated with AD and can be observed in all stages of the disease (Gillette-Guyonnet et al., 2000; Guérin et al., 2005; Cova et al., 2016). Weight loss in AD patients often correlates with a general health decline and is suggested as a clinical predictor of mortality (White et al., 1998). In mice, weight variances can also indicate poor health and a lack of body weight gain might contribute to predicting mortality.

In contrast to 5XFAD mice, Tg4-42 mice did not show a limb clasping phenotype. Aged 5XFAD mice exhibited motor abnormalities and abnormal extension reflexes by retracting their hind- and fore paws simultaneously when suspended by the tail. An abnormal clasping pattern has been observed in several mouse models including mice transgenic for human four-repeat tau and mutant human APP (Probst et al., 2000; Lalonde et al., 2012), as well as APP/PS1KI and 5XFAD mice. Interestingly, all of these mice showed signs of axonopathy (Tesseur et al., 2000; Wirths et al., 2007; Jawhar et al., 2012). But surprisingly, the number of motor neurons of 6-month-old 5XFAD mice in the cervical spinal cord was similar to wild-type littermates (Chu et al., 2017). Both Tg4-42 and 5XFAD mice show intracellular Aβ accumulation in the spinal cord, but only 5XFAD show signs of axon swelling and axonopathy (Jawhar et al., 2012; Lopez-Noguerola et al., 2018).

The Open Field locomotion test can be used to examine motor function by measuring spontaneous activity in an open arena (Lee et al., 2009). The Open Field task is an easy task to perform and measures general locomotor activity in mice (Seibenhener and Wooten, 2015). Our results show that Tg4-42 mice did not have alterations in locomotor activity. In contrast, 5XFAD mice showed lower activity levels consistent with previous studies (Sawmiller et al., 2017). Surprisingly, O’Leary et al. (2018a) found increased locomotor activity in 6- to 7-month-old female 5XFAD mice. This variation is likely due to different experimental protocols. However, in older 5XFAD mice the data is consistently showing reduced locomotor activity (Schneider et al., 2014; Griñán-Ferré et al., 2016; O’Leary et al., 2018a,b) (Table 1). Altered locomotor activities need to be taken into consideration when interpreting results of behavioral tests such as anxiety-related or memory-related tests (Crawley and Bailey, 2008; Brooks et al., 2012; Deacon, 2013). Nevertheless, Tg4-42 mice did not show any impairments in the Open Field that could possibly influence anxiety- and memory-related behavior. In line with these findings, general motor abilities of Tg4-42 mice have been shown to be intact in the Morris water maze as swimming speed did not differ from wild-type animals (Bouter et al., 2013, 2014, 2019; Antonios et al., 2015).

Generally speaking, APP single-transgenic mice seem to show increased locomotor activity at young ages (King and Arendash, 2002; Harris et al., 2010), whereas mouse models with multiple mutations develop impairments at an older age (Gulinello et al., 2009; Filali et al., 2012; O’Leary et al., 2018a). Similar to Tg4-42 mice, double-transgenic mice carrying single APP and PS1 mutation are less likely to have locomotor activity impairments (Lalonde et al., 2004; Sadowski et al., 2004; Webster et al., 2013) (Table 1).

It has to be mentioned that the Open Field task is one of the tests with relatively high variability in experimental procedures (Walsh and Cummins, 1976). The material and the size of the arena, as well as the testing time, differ immensely between the protocols used in analyzing different AD models. For example, some mice were granted time to adapt to the apparatus (van Dam et al., 2003) and others not (Lalonde et al., 2005a; Harris et al., 2010; Jawhar et al., 2012). The amount of time tested ranged from 5 min (Arendash et al., 2001; Jawhar et al., 2012; O’Leary et al., 2018a) to 30 min (Dineley et al., 2002; Webster et al., 2013). In addition, most mice were placed into the center of the box (King and Arendash, 2002; Jawhar et al., 2012), whereas others started in the corners (van Dam et al., 2003; O’Leary et al., 2018a). While most studies use only a single trial, other groups tested the same mouse multiple times and therefore assessing habituation rather than spontaneous locomotor activity alone (Dai et al., 1995; Lalonde et al., 2005b). Furthermore, the experimental apparatus used for the Open Field often differs in size, material, form or transparency between laboratories (King and Arendash, 2002; Gulinello et al., 2009; Filali et al., 2012; Jawhar et al., 2012). These varieties need to be taken into consideration when comparing different AD mouse lines.

Several studies suggested a link between muscle strength and cognitive decline in AD patients (Boyle et al., 2009). In mice, the string suspension and grip strength test can be used to measure muscle strength (Deacon, 2013). We could show that 7-month-old Tg4-42 and 5XFAD mice did not present any impairments in muscle strength in the string suspension or grip strength tasks, consistent with previous findings for 5XFAD mice (Jawhar et al., 2012; Bhattacharya et al., 2014). However, previous studies showed a loss of muscle strength in both tests beginning at the age of 9 months (Jawhar et al., 2012). In contrast, O’Leary et al. (2018a) could show deficits already beginning from 6 to 7 months of age in 5XFAD mice. Generally speaking, a decline in muscle strength seems to be more frequent in aged AD mice (Table 1). Tg4-42 mice display severe memory deficits at 7 months of age and therefore mice were not tested for muscle strength at older ages, which should be investigated in upcoming studies to compare strength with other AD mouse models. The pyroglutamate Aβ3-42 expressing mouse model TBA42 showed muscle strength deficits starting with 6 months, while muscle strength declined in Tg2576 not before 14 months, preceding memory deficits in both models (King and Arendash, 2002; Meissner et al., 2014) (Table 1). Interestingly, homozygous TBA42 had such severe motor deficits, that they had to be sacrificed at the age of 2 months (Lopez-Noguerola et al., 2018). APP+PS1 mice did not show signs of strength decline before 15 to 17 months of age (Holcomb et al., 1999; Arendash et al., 2001). APP+PS1 mice develop memory deficits at younger ages, similar to 5XFAD mice. Noteworthy, female and male 3xTg mice show memory impairments before the onset of motor deficits with 16 months of age (Garvock-de Montbrun et al., 2019), similar to Tg4-42 mice (Table 1), but memory deficits do not progress through age in 3xTg mice (Stevens and Brown, 2015). These inconsistent findings with regard to onset of memory and motor deficits should be considered when choosing a suitable mouse model for research, depending on the symptom of interest.

Motor coordination is an important aspect of AD since bradykinesia and gait disturbances are part of AD motor impairments (Scarmeas et al., 2004), correlating with a poorer outcome of patients (Waite et al., 2005).

The rotarod and balance beam test are mainly used to test motor coordination and balance in mice. Additionally, the rotarod test assesses motor learning (Hamm et al., 1994; Le Marec and Lalonde, 1997; Luong et al., 2011; Deacon, 2013).

The cerebellum as a major player of motor control is important for regulation of balance and locomotion through practice (Morton and Bastian, 2004). Especially the rotarod task is able to detect motor disturbances through cerebellar dysfunction (Shiotsuki et al., 2010).

We could show deficits in the balance beam task of aged Tg4-42 mice. In context with Lopez-Noguerola et al. (2018) who tested younger Tg4-42 mice at the age of 5 months, an age dependent decline in balance and coordination could be observed. These findings suggest an onset of this deficit in the Tg4-42 mouse model for AD at the age of 6 to 7 months.

Deterioration in the rotarod test was already observed in young Tg4-42 mice and aggravated with age. Early deficits in the rotarod have been described in male APP23 mice (van Dam et al., 2003). In contrary Tg2567 mice did not show any impairment in the rotarod (Dineley et al., 2002; King and Arendash, 2002), but the balance beam test was able to detect deficits (King and Arendash, 2002). APP/PS1 KI mice did not show any motor deterioration and were comparable to controls at even older ages (Webster et al., 2013). Strikingly, male and female 3xTg mice performed better in rotarod task than controls up to the age of 16 months (Oore et al., 2013; Stover et al., 2015; Garvock-de Montbrun et al., 2019) (Table 1), but performance declined with age (Garvock-de Montbrun et al., 2019). Only Filali et al. (2012) who tested only female 3xTg mice showed reduced performance in the rotarod, possibly due to protocol differences. Stover et al. (2015) suggests that tau P301L might be responsible for enhanced motor performance. Whereas other researchers found increased motor performance to be paradoxical (Filali et al., 2012), since other tau mouse models do present motor impairments in the rotarod test (Scattoni et al., 2010; Xu et al., 2010). Thus, suggesting no direct correlation between the transgene and severity of motor deficits.

Since Tg4-42 mice did not have altered muscle strength, we presumed that impairments in the balance beam task and rotarod was rather due to learning and balance, coordination impairments than muscle function. In previous studies we could show a severe learning deficit in the Morris water maze, beginning from the age of 5 months in Tg4-42 mice (Antonios et al., 2015). Interestingly, despite the massive impairments in the rotarod, the ability to swim in the Morris water maze was not altered in same-aged Tg4-42 mice (Bouter et al., 2013, 2014; Antonios et al., 2015).

The cerebellum is the center of motor function and contributes to motor learning by determining how to perform correct and accurate movements. It has been shown that cerebellar damage leads to disturbance in movements and body support (Porras-García et al., 2013) and cerebellar atrophy is characteristic for sporadic AD (Jacobs et al., 2018). Kuwabara et al. (2014) could detect relatively high levels of Aβ1-42 in cerebellar lysates and impairments in the rotarod test in 3-month-old APP/PS1 mice, but no impairments in the balance beam test.

Subsequently, we analyzed the cerebellum of Tg4-42 mice via ^18^F-FDG-PET to detect possible synaptic dysfunctions. ^18^F-FDG-PET, as a functional biomarker for synaptic dysfunction, confirmed findings of the behavioral tests showing reduced glucose metabolism in the cerebellum of aged Tg4-42 mice. Findings are in line with several earlier ^18^F-FDG-PET studies showing cerebellar hypo metabolism in different transgenic mouse models of AD (Platt et al., 2011; Macdonald et al., 2014; Deleye et al., 2016; Takkinen et al., 2017; Waldron et al., 2017; Bouter et al., 2019). But furthermore, intraneuronal Aβ accumulation in the spinal cord of Tg4-42 mice have been described previously (Lopez-Noguerola et al., 2018) and may contribute to motor deficits. But since strength was not yet affected in 7-month-old Tg4-42 mice we focused on the processing and coordination of motor function by *in vivo* imaging of the cerebellum.

Interestingly, a study by Macdonald et al. (2014) using ^18^F-FDG-PET in 2-, 5-, and 12-month-old 5XFAD mice showed decreased FDG-uptake in the cerebellum of 12-month-old 5XFAD mice while 2- and 5-month-old animals did not show significant differences to wild-type mice. In line with these findings, female 7-month-old 5XFAD performed as wild-type mice on the rotarod, while older mice display strong impairments (O’Leary et al., 2018a) (Table 1). However, 5XFAD mice showed decreased FDG-uptake as early as 7 months of age in our study. This seems to be an indicator of early cerebellar changes, even before motor impairments occur, as glucose metabolism is known to be an early marker of neuronal dysfunction.

Mouse models allow to investigate motor abilities and are suitable tools to analyze different aspects of AD pathologies and the effectiveness of possible therapies.

Tg4-42 displays an AD mouse model with intact general motor activity and strength, but age-dependent motor impairments in motor coordination and balance present at the age of 7 months, most likely due to impaired cerebellar activity.

## Data Availability

The datasets generated for this study are available on request to the corresponding author.

## Ethics Statement

All animals were handled according to the guidelines of the “Society for Laboratory Animals Science” (GV-SOLAS) and the guidelines of the “Federation of European Laboratory Animal Science Association” (FELASA). All experiments were approved by the “Lower Saxony State Office for Consumer Protection and Food Safety” (LAVES).

## Author Contributions

JW performed the experiments, analyzed the data, and wrote the manuscript. MS performed the experiments and analyzed the data. ES, ML, TF, NB, and CI performed the experiments. CB analyzed the data and wrote the manuscript. TB participated in the discussion of the results. YB conceived and designed the project, performed the experiments, analyzed the data, and wrote the manuscript. All authors contributed to revising the manuscript and approved the final version.

## Conflict of Interest Statement

University Medicine Göttingen has been granted a patent on the Tg4-42 mouse model, on which TB is listed as an inventor. The remaining authors declare that the research was conducted in the absence of any commercial or financial relationships that could be construed as a potential conflict of interest. The reviewer SV declared a past collaboration with one of the authors TB to the handling Editor.
